# ZooKeys anniversary: 10 years of leadership toward open-access publishing of zoological data and establishment at Pensoft of like-minded sister journals across the biodiversity spectrum

**DOI:** 10.3897/zookeys.770.28105

**Published:** 2018-07-04

**Authors:** Terry Erwin, Pavel Stoev, Lyubomir Penev

**Affiliations:** 1 National Museum of Natural History, Smithsonian Institution, Washington DC, USA; 2 National Museum of Natural History, Bulgarian Academy of Sciences, Sofia, Bulgaria; 3 Pensoft Publishers, Sofia, Bulgaria; 4 Institute of Biodiversity and Ecosystem Research, Bulgarian Academy of Sciences, Sofia, Bulgaria

Today we publish issue 770 of our dear cutting-edge journal ZooKeys! It has been exactly ten years since the launch of the journal on 4 July 2008 that emanated from a delightful breakfast at the Entomological Society of America meeting in December 2007 in San Diego, California, when our Managing Editor and founder of Pensoft, Lyubomir Penev, proposed the idea to Terry Erwin, our Editor-in-Chief. The journal’s tenth birthday is a great occasion to trace back its development and achievements since then, which has exceeded far beyond that initial breakfast dream of two colleagues enjoying the southern California sun.

**Figure 1.**
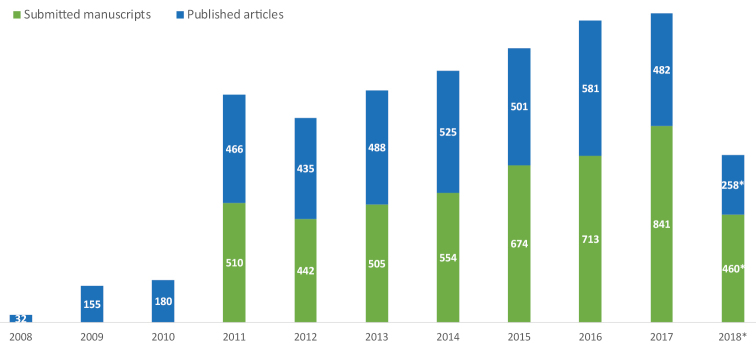
Growth of submitted manuscripts and published articles in ZooKeys from 2008 to 2018 (*until 27.6.2018).

ZooKeys was the first of Pensoft’s open-access journals, set up to accelerate research and free information exchange in taxonomy, phylogeny, biogeography and evolution of animals. Starting as a taxonomic journal, it quickly expanded to other zoology-related sciences, such as ecology, molecular biology, genomics, evolutionary biology, palaeontology, behavioural science, bioinformatics etc. Later, ZooKeys was followed by the journals PhytoKeys and MycoKeys in the field of plant and fungal systematics, which are now also amongst the most popular titles in their respective domains. The journal has been thriving since its inception and is currently considered as one of the most prolific and liked Open Access journals in zoology. ZooKeys started with merely 32 published papers in 2008 and just in a few years time became a mega-journal, publishing 466 papers in 2011. The number has been increasing since reaching its maximum in 2016–581 (Table 1, Fig. 1). To date, the journal has received more than 5200 submissions (no accurate data available for 2008–2010) and published 4103 articles, including 110 monographs. The number of published pages increased from 657 in 2008 to 16582 in 2016. The average rejection rate for the period 2016–2017 was around 25%, which we believe is optimal and sustainable for a primarily taxonomic journal.

**Table 1.** Total number of submitted manuscripts published articles, and printed pages since 2008. No accurate data for number of submissions 2008–2010.

**Year**	**Submitted manuscripts**	**Published articles**	**Published pages**
2008		32	657
2009		155	3738
2010		180	4871
2011	510	466	11145
2012	442	435	12205
2013	505	488	13382
2014	554	525	14178
2015	674	501	12634
2016	713	581	16582
2017	841	482	14091
2018 (as of 27 June 2018)	460	258	7250
**Total**	**4904**	**4103**	**110733**

The number of all authors publishing in ZooKeys is 5720 (ZooBank, courtesy of Richard Pyle, Bishop Museum, Honolulu) from altogether 131 countries. The highest numbers come first from China, then United States of America, followed by Brazil, Italy, Germany and Canada in that order. The Impact Factor of ZooKeys continues to grow, starting at 0.517 and currently it is 1.079.

**Figure 2.**
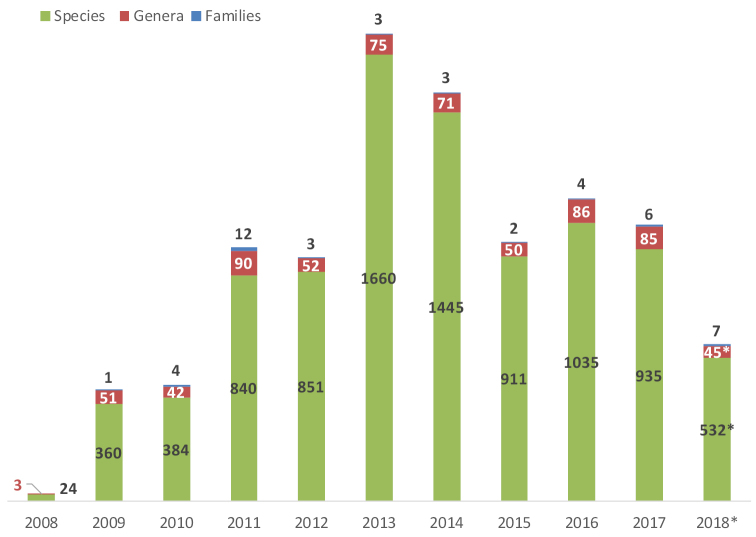
New taxa published in ZooKeys, registered in ZooBank (courtesy of Richard Pyle; *until 27.6.2018).

Altogether, 8977 new species-group, 650 new genus-group and 45 new family-group taxa have been published in the journal since its launch (Table 2, Fig. 2) (ZooBank, 29 June 2018, courtesy of Richard Pyle). This makes 9672 new taxa in total or 967.2 new taxa per year. This places ZooKeys as the second most prolific journal in Zoological Systematics after Zootaxa which began publishing in 2001.

**Table 2.** New taxa published in ZooKeys, registered in ZooBank (courtesy of Richard Pyle).

**Year**	**Family**	**Genus**	**Species**
2008	0	3	24
2009	1	51	360
2010	4	42	384
2011	12	90	840
2012	3	52	851
2013	3	75	1660
2014	3	71	1445
2015	2	50	911
2016	4	86	1035
2017	6	85	935
2018	7	45	532
**Total**	**45**	**650**	**8977**

Pensoft has been heavily investing in the technological advancement of its journals. A list of the most significant technologies implemented by its flagship ZooKeys in the recent years to facilitate editors, reviewers and authors is available in Table 3.

**Table 3.** New technological solutions implemented in the journal.

Feature	For the benefit of:	Link	Use
Automatic registrations of reviews at Publons	Reviewers and Editors	https://publons.com	Publons helps reviewers and editors get recognition for every review they make for the journal.
Dimensions	Authors, editors, administrators, publisher	https://www.dimensions.ai	Powerful tracker of citations; provides ranking of given research in a given field
Scopus CiteScore Metrics	Authors, editors, administrators, publisher	https://www.scopus.com/sourceid/19700170477	Interactive tool providing information on journal’s performance
Export of published figures & supplementary materials to Biodiversity Literature Repository at ZENODO	Authors, data scientists, community in general	https://zenodo.org/communities/biosyslit/?page=1&size=20	Increases visibility and traceability of article and sub-article elements
Hypothes.is	Authors, readers	http://hypothes.is	Annotations on selected texts from the published article

Over the past ten years, ZooKeys published a variety of papers on systematic zoology, including several world records, such as the deepest cave-dwelling centipede, the tiniest free-living insect and the smallest land snail. The journal also served as a platform for many of the world’s first-of-a-kind, like the first insect description solely from photographs, the first study supported by crowd-funding in Japan, the first-of-a-kind footage of shrimp filter-feeding at depth of a 4826 m in the Mariana Trench, the first amphibious centipede and the second fossil beetle found on Antarctica. While ZooKeys is regularly featured in the annual “Top 10 species” by the International Institute for Species Exploration, in 2017, there were two species published in the journal, which appeared on the list: the world’s second leggiest millipede – the 414-legged *Illacme tobini* and the first known amphibious centipede *Scolopendra cataracta*.

The ten most viewed ZooKeys articles can be seen in Table 4.

**Table 4.** ZooKeys articles by number of views.

**Article**	**Nr of uniques views**	**Nr of total views**
Helgen et al. (2013) Taxonomic revision of the olingos (*Bassaricyon*), with description of a new species, the Olinguito	56191	62724
Ledford et al. (2012) An extraordinary new family of spiders from caves in the Pacific Northwest (Araneae, Trogloraptoridae, new family)	51668	55952
Bouchard et al. (2011) Family-Group Names In Coleoptera (Insecta)	32446	36687
Nazari (2016) Review of *Neopalpa* Povolný, 1998 with description of a new species from California and Baja California, Mexico (Lepidoptera, Gelechiidae)	24654	33103
Sereno (2012) Taxonomy, morphology, masticatory function and phylogeny of heterodontosaurid dinosaurs	27168	30394
Hagedorn et al. (2011) Creative Commons licenses and the non-commercial condition: Implications for the re-use of biodiversity information	27173	29685
Winterton et al. (2012) A charismatic new species of green lacewing discovered in Malaysia (Neuroptera, Chrysopidae): the confluence of citizen scientist, online image database and cybertaxonomy	25329	28429
Laciny et al. (2018) *Colobopsis explodens* sp. n., model species for studies on “exploding ants” (Hymenoptera, Formicidae), with biological notes and first illustrations of males of the *Colobopsis cylindrica* species-group	22795	28258
Hamilton et al. (2016) Taxonomic revision of the tarantula genus *Aphonopelma* Pocock, 1901 (Araneae, Mygalomorphae, Theraphosidae) within the United States	14515	25536
Wizen and Gasith (2011) Predation of amphibians by carabid beetles of the genus *Epomis* found in the central coastal plain of Israel	24477	14876
**Total**	**281087**	**282920**

**Figure 3.**
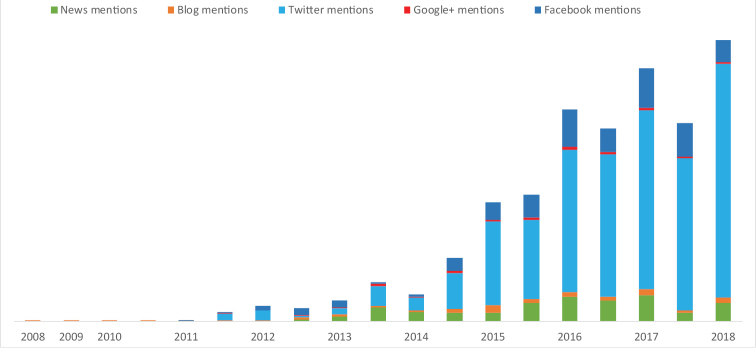
Total number of ZooKeys mentions in social media and popular magazines (Altmetric, June 2018).

Thanks to the collaboration between Pensoft and Altmetric, it is possible to track the popularity of each article published in ZooKeys within the public domain (Fig. 3). Provided the DOI link of a paper is included in an online publication, its citations from across a diverse range of both conventional and social online media platforms, including news outlets, blogs, Twitter, Facebook, Google+, Reddit etc., are all visible in the article menu to give our readers a clear insight into the attention and interest which the research published in the journal brings about beyond academia.

The description of a species of moth named after then US President-elect Donald Trump is an excellent example for a study with remarkable popularity across platforms (available from https://doi.org/10.3897/zookeys.646.11411). While as many as 964 tweets have been registered (likely many more, given that the count only registers the tweets featuring the DOI link to the paper), a total of 124 international news outlets (again, many have gone unaccounted) ran the story, including The Washington Post, FOX News, CNN, BBC News, The Independent, The Huffington Post, Ria.RU (RIA Novosti), Gazeta.ru, Wired (Italy), Le Figaro, Die Welt, Spiegel, National Geographic Australia, The Japan Times and The Hindustan Times.

A more recent study describing a new species of exploding ant was not only featured in 89 news stories by news outlets from around the world, such as National Geographic, The New York Times, FOX News, BBC News, Sky News, The Guardian, ABC, Gazeta.ru, Publico, Stern, El Pais, The Hindu, but also tweeted along with its DOI as many as 52 times. In fact, the remarkable species was even ‘assigned’ with its own hashtag (#ExplodingAnts) to trigger further discussion and engagement over the social media platform.

Table 5 shows the top ten ZooKeys papers, which attracted the largest media interest, according to data available from the global science news service Eurekalert.

**Table 5.** The top ten ZooKeys papers that attracted largest media interest.

Article	Press release	Media coverage
Brannoch and Svenson (2016) A new genus and species (*Cornucollis* gen. n. *masoalensis* sp. n.) of praying mantis from northern Madagascar (Mantodea, Iridopterygidae, Tropidomantinae)	A new species and genus of ‘horned necked’ praying mantis from a French museum collection	Science Daily, Physorg, Health Medicine Network
Chen et al. (2017) *Oreoglanis hponkanensis*, a new sisorid catfish from north Myanmar (Actinopterygii, Sisoridae)	Chinese scientists discover a new species of catfish in Myanmar	Science Newsline, Physorg, Health Medicine Network, I4U News
Laciny et al. (2018) *Colobopsis explodens* sp. n., model species for studies on “exploding ants” (Hymenoptera, Formicidae), with biological notes and first illustrations of males of the *Colobopsis cylindrica* species-group	New ant species from Borneo explodes to defend its colony	New York Times, The Guardian, Galileo, Gazeta.ru, New York Daily News
Van Dam et al. (2016) Four new species of *Trigonopterus* Fauvel from the island of New Britain (Coleoptera, Curculionidae)	New curiously scaled beetle species from New Britain named after ‘Star Wars’ Chewbacca	The Scientist Magazine, Fox News, Science News
Savary and Bryson Jr (2016) *Pseudouroctonus maidu*, a new species of scorpion from northern California (Scorpiones, Vaejovidae)	A new scorpion from California reveals hidden biodiversity in the Golden State	Science Daily, Physorg, Health Medicine Network
Hamilton et al. (2016) Taxonomic revision of the tarantula genus *Aphonopelma* Pocock, 1901 (Araneae, Mygalomorphae, Theraphosidae) within the United States	New tarantula named after Johnny Cash among 14 spider species found in the United States	CNN, BBC News, CBS News, The Guardian, The Columbian, Spiegel, Gazeta.ru
Guayasamin et al. (2017) A marvelous new glassfrog (Centrolenidae, Hyalinobatrachium) from Amazonian Ecuador	New species of frog from the Neotropics carries its heart on its skin	BBC Focus Science & Technology, Science News, Gazeta.ru, Science Daily
Nazari (2017) Review of *Neopalpa* Povolný, 1998 with description of a new species from California and Baja California, Mexico (Lepidoptera, Gelechiidae)	New species of moth named in honor of Donald Trump ahead of his swearing-in as president	CNN, CBS News, The Straits Times, The Independent, Gazeta.ru, Focus, Galileo
Goto and Ishikawa (2016) *Borniopsis mortoni* sp. n. (Heterodonta, Galeommatoidea, Galeommatidae sensu lato), a new bivalve commensal with a synaptid sea cucumber from Japan	Living together in mud: New bivalve species dwelling on a sea cucumber discovered in Japan	Nature World News, Health Medicine Network, Physorg
Marek et al. (2016) A new species of *Illacme* Cook & Loomis, 1928 from Sequoia National Park, California, with a world catalog of the Siphonorhinidae (Diplopoda, Siphonophorida)	New species of extremely leggy millipede discovered in a cave in California	New York Times, Washington Post, Gizmodo, Nature World News, Le Point

Apart from their remarkable findings, some of our authors have also been given a place in the spotlight by the news media. A Skype interview with Dr Chris Hamilton – the discoverer of the Johnny Cash tarantula – was aired live on Sky News, while Dr Vazrick Nazari, who added the name *Neopalpa donaldtrumpi* to the scientific records, was interviewed on BBC Radio 5. A podcast with Alice Laciny, the lead author of the study describing the exploding ant *Colobopsis explodens*, where she explains the curious behaviour of the new species and in the background, the ant is seen to actually defend itself against a larger offender, was made available on BBC News.

New species described in ZooKeys enjoy the attention of their celebrity namesakes, as well. Earlier this year, shortly after a water beetle discovered in Borneo was named after the famous actor and environmentalist Leonardo DiCaprio, the insect appeared on his profile photo on Facebook – an act, which was itself reported by several news outlets, including the Daily Mail, W Magazine and La Republica.

**The success of ZooKeys would not be possible without the help of our authors, reviewers, subject editors, and readers, to whome we are very very thankful**!
